# A Case of Cytomegalovirus-Induced Hemophagocytic Lymphohistiocytosis in a Patient with an Underlying Rheumatic Disease

**DOI:** 10.7759/cureus.8130

**Published:** 2020-05-15

**Authors:** Arthur Lau, Hayoung Youn, Roberto Caricchio, Lawrence Brent

**Affiliations:** 1 Rheumatology, Temple University Hospital, Philadelphia, USA

**Keywords:** hemophagocytic lymphohistiocytosis, cytomegalovirus-cmv, secondary hlh, inflammatory myositis, anakinra, etoposide, azathioprine, immunosuppressed, macrophage activating syndrome, mas

## Abstract

Hemophagocytic lymphohistiocytosis (HLH) is a life-threatening condition caused by overproduction of inflammatory cytokines and overactivation of macrophages that can progress to multiorgan dysfunction and failure. Although there are guidelines that attempt to recognize the condition in its early stage, diagnosis can be very challenging due to heterogeneous presentations of HLH. Symptoms and clinical findings include fever, neurologic complaints, respiratory issues, liver dysfunction, cytopenias, amongst others most of which are not specific to HLH. In addition, response to treatment can be highly variable, necessitating an individualized treatment plan based on the presentation. We present a case of a 21-year-old female with a history of biopsy-proven inflammatory myositis on azathioprine and prednisone who presented with fever, hypotension, and pancytopenia. Additional imaging studies showed multiorgan involvement, including pneumonia, pyelonephritis, and splenomegaly. A bone marrow biopsy of her iliac crest showed hemophagocytosis and the infectious workup confirmed cytomegalovirus (CMV) infection, which led to the diagnosis of CMV-induced HLH. She was treated initially with anakinra for macrophage activation syndrome (MAS) in addition to dexamethasone and ganciclovir. Unfortunately, she did not respond to anakinra and was subsequently switched to etoposide with dexamethasone and valganciclovir, which subsequently helped our patient to recover clinically. Our case highlights the challenging nature of HLH and the importance of early detection and a personalized treatment plan in achieving optimal outcomes in patients with HLH.

## Introduction

Hemophagocytic lymphohistiocytosis (HLH) is a potentially life-threatening condition that leads to multiorgan involvement and organ failure due to overactivation of the immune system. The aberrant immune system leads to the production of increased inflammatory cytokines and macrophage activation resulting in systemic symptoms and signs [1-2]. These signs are variable from patient to patient and may be nonspecific, making the diagnosis of HLH a diagnostic challenge for clinicians. Recognizing HLH early is important as a delay in treatment may lead to death with overall mortality ranging from 20% to 88%, depending on patient characteristics and follow up [3]. Diagnosis can be made through genetic testing or based on clinical diagnostic criteria. Treatment is directed at identifying and treating any underlying trigger, suppressing inflammation, or by destroying immune cells [1]. We report a case of cytomegalovirus (CMV)-induced hemophagocytic lymphohistiocytosis (HLH) in an immunosuppressed patient with inflammatory myositis treated with anakinra and subsequently etoposide in combination with dexamethasone and antiviral agents.

## Case presentation

**Figure 1:**
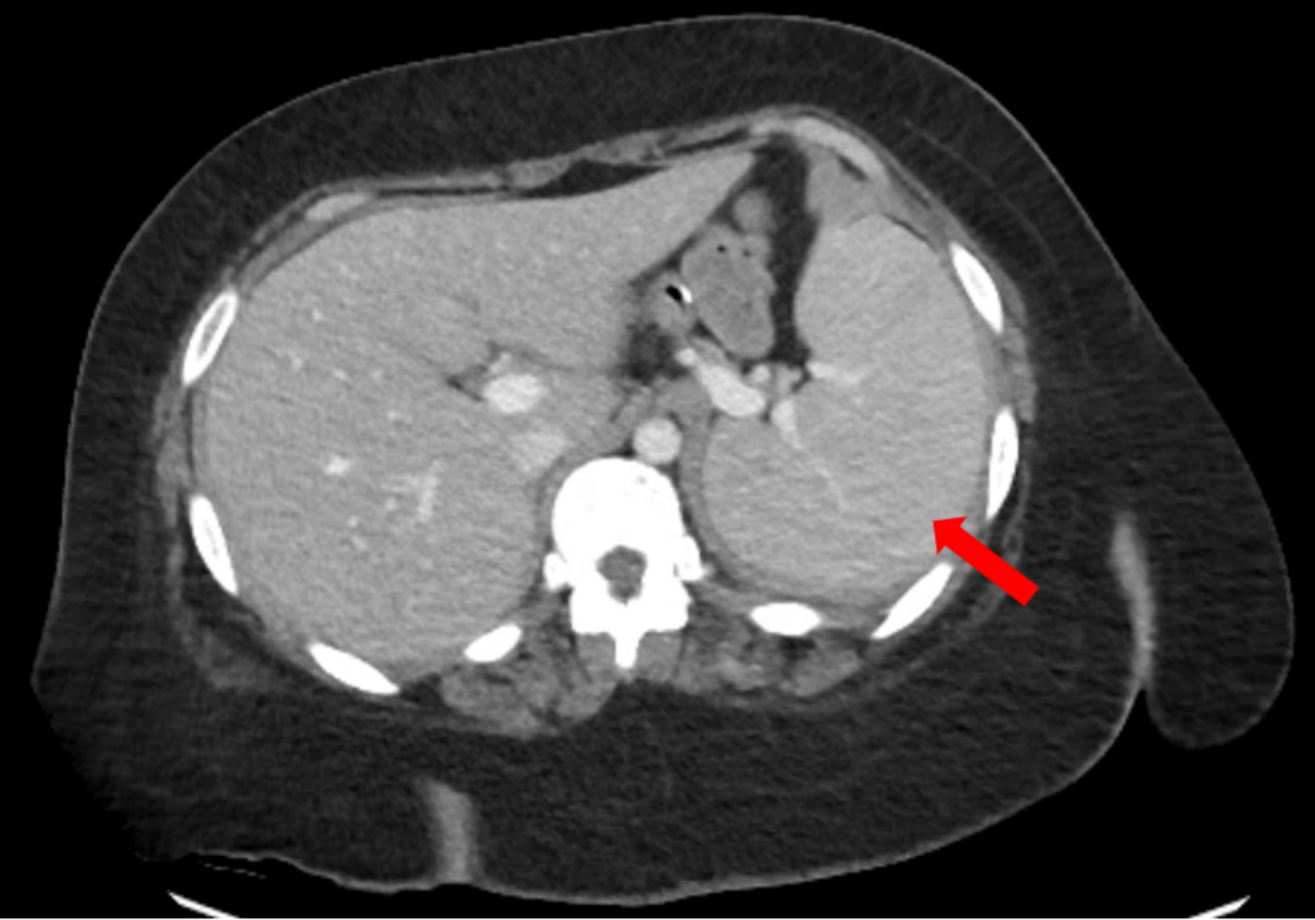
CT scan of the abdomen showing evidence of splenomegaly (red arrow).

A 21-year-old female with a past medical history of inflammatory myositis on prednisone and azathioprine, gastric bypass, and morbid obesity presented to the rheumatology outpatient clinic with subjective fevers and shortness of breath. She carried a history of biopsy-proven inflammatory myositis diagnosed two years earlier and treated initially with high dose steroids and rituximab with subsequent improvement, currently maintained on azathioprine 200 mg oral daily and prednisone 20 mg oral daily. Initial exam revealed coarse rhonchi, diaphoresis, somnolence, and worsening proximal muscle weakness. Laboratory tests performed two days earlier showed pancytopenia with white blood count (WBC) of 0.83 µL, hemoglobin (Hgb) of 9.7 g/dL, platelets of 76000 µL, and 14% bands. She was sent immediately to the ER for evaluation where she was found to be tachycardic at 108 bpm, and hypotensive with a blood pressure of 81/41 mmHg. Additional laboratory results obtained showed evidence of an elevated lactate of 2.2 mmol/L, creatinine kinase of 581 U/L, aldolase of 32.1 U/L, ferritin of 3300 ng/mL as well as abnormal liver function tests. A chest X-ray showed patchy airspace opacification in the left perihilar region suggestive of pneumonia. She subsequently had a CT scan of the chest which showed ground glass opacities suggestive of atypical pneumonia, in addition to a CT of her abdomen which showed possible left sided pyelonephritis and splenomegaly (Figure 1).

The patient was started on broad spectrum antibiotics including vancomycin, piperacillin/tazobactam, and azithromycin and she was given IV fluids with stabilization of her blood pressure. Azathioprine 200 mg oral daily was discontinued and she was initiated on stress dose steroids with hydrocortisone 100 mg IV q6 hours. Rheumatology, hematology, and infectious disease were consulted to evaluate the patient at this time.

**Figure 2:**
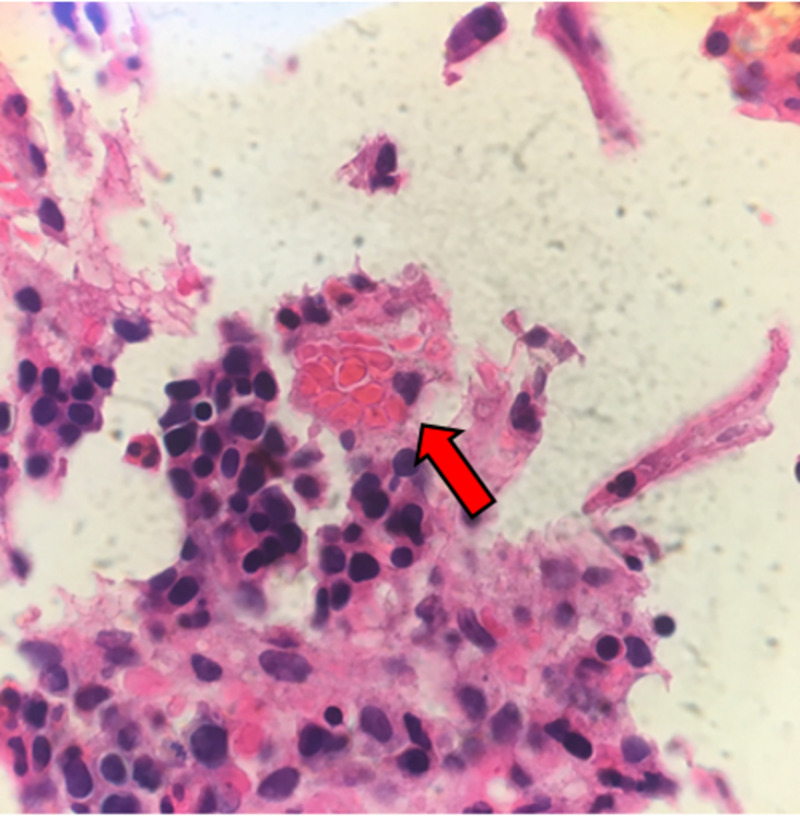
Scattered macrophages with phagocytosed red blood cells and other cell debris consistent with hemophagocytosis.

Given the patient’s history of inflammatory myositis, pancytopenia, splenomegaly, elevated ferritin, and fever, a suspicion for HLH was raised. Further diagnostic workup revealed a low fibrinogen of 66 mg/dL, elevated triglycerides of 259 mg/dL, and elevated soluble IL-2R (CD25) of 8942 pg/mL. A bone marrow biopsy was ultimately performed from the patient’s right iliac crest showing evidence of hemophagocytosis, thus confirming a diagnosis of HLH (Figure 2). There was no evidence of malignancy from her bone marrow biopsy and no genetic testing was performed.

Infectious workup eventually revealed a positive test for CMV IgM, and her CMV viral load was elevated at 740,862 IU/mL copies. The patient was diagnosed with CMV-induced HLH and subsequently started on ganciclovir 500 mg IV q12 hours (5 mg/kg q12 hours dose adjusted for body weight). After a multidisciplinary meeting, the patient was started on anakinra 100 mg subcutaneously three times daily and dexamethasone 25 mg IV daily to treat the HLH. Anakinra was chosen over etoposide due to its more favorable safety profile and to minimize further significant immunosuppression in the setting of infection. Although the patient tolerated the anakinra, she did not respond to the medication despite seven days of therapy as she continued to have persistently elevated ferritin levels, pancytopenia, worsening liver function tests, and elevated bilirubin levels. Anakinra was stopped and the patient was switched to etoposide 75 mg/m2 using the HLH-94 protocol (dose reduced due to poor hepatic function and elevated bilirubin levels). A total of three doses of etoposide were given with a good response including improvement of her liver function tests, bilirubin levels, and ferritin. However, after the third dose, she remained severely neutropenic with worsening absolute neutrophil count (ANC) and new fevers that ultimately led to the etoposide being stopped. Blood cultures grew Klebsiella pneumoniae and she was started on cefepime and vancomycin for febrile neutropenia. Dexamethasone dosage was reduced from 25 mg IV to 15 mg IV daily and ganciclovir was transitioned to foscarnet due to concerns that the ganciclovir was contributing to her cytopenia. The foscarnet led to persistent electrolyte abnormalities that required aggressive repletion of her potassium and calcium, and thus, the decision was made to transition the patient to valganciclovir 900 mg oral BID. During this time, the patient’s ANC began to recover as her CMV viral load decreased to 291 IU/mL. The patient also had improvement of her liver function tests, bilirubin levels, cytopenia in addition to ferritin level. She was discharged on CMV therapy with slow taper of her corticosteroids and close follow up with hematology, infectious disease, and rheumatology. On outpatient follow up, the patient was doing well clinically with reduction of her CMV viral load to <200 IU/mL, and normalization of the complete blood count and ferritin level.

## Discussion

Hemophagocytic lymphohistiocytosis is a life-threatening condition characterized by a dysregulated immune system leading to excessive production of inflammatory cytokines, multiorgan failure, and significant mortality [1]. HLH was first described in 1939 by Scott and Robb-Smith in four patients in which postmortem exam revealed histiocytes phagocytosing erythrocytes [2]. Several years later in 1952, Farquhar and Claireaux described the disease as a rare familial disorder due to a proliferation of histiocytes and phagocytosis of blood cells [4-5]. HLH is often classified as primary HLH seen typically in children with an underlying genetic disorder, or secondary HLH which is due to another underlying condition (e.g. infection, malignancy) [4]. If the secondary condition is due to a rheumatologic cause (e.g. systemic onset juvenile idiopathic arthritis, systemic lupus erythematosus, vasculitis, inflammatory myositis), HLH is referred to as macrophage activation syndrome (MAS) [6]. HLH is seen in all age ranges and has no specific predilection for sex or race [4].

Hemophagocytic lymphohistiocytosis is a hyperinflammatory immune response syndrome leading to activation of a cascade of inflammatory pathways causing cytokine storm. This leads to nonspecific clinical manifestations including fever, neurologic findings, respiratory issues, coagulopathy, liver dysfunction, cytopenias, hyperferritinemia, amongst others [1]. Particularly in MAS, excessive activation and expansion of T lymphocytes and macrophages exhibiting hemophagocytic activity lead to cytokine overproduction, tissue damage, and organ failure. Cytokines that have been implicated in the pathogenesis include IL-1, IL-6, Il-18, TNFα, and IFNγ [6-7]. This cycle is perpetuated due to the absence of downregulation of activated macrophages and lymphocytes due to impaired function of natural killer (NK) cells or cytotoxic T lymphocytes [6]. Overall, this leads to the immune system being overactive but ineffective in responding to an inciting stimulus. Treatment is directed at breaking this vicious cycle by downregulating the immune response [1].

Hemophagocytic lymphohistiocytosis remains a diagnostic challenge to identify early unless a high index of suspicion is maintained as it may mimic other inflammatory syndromes including sepsis and malignancy. Often, many of the initial tests that are helpful in evaluating HLH may have already been performed as part of the evaluation of multiorgan failure in a febrile patient. Even more challenging in adults, evidence for diagnosis and treatment of HLH primarily comes from the pediatric literature [8]. The diagnosis of HLH can either be made with molecular testing or if the patient fulfils at least five of the following eight diagnostic criteria listed in Table 1. Our patient met seven of the eight criteria based on the Histiocyte Society proposed diagnostic criteria from 2004.

**Table 1:** Diagnostic guidelines for HLH [2, 8]. *Our patient met seven of the eight clinical diagnostic criteria. HLH, hemophagocytic lymphohistiocytosis

Diagnostic guidelines for HLH
Molecular diagnosis or 5/8 diagnostic criteria below:
1. Fever*
2. Splenomegaly*
3. Cytopenias (affecting ≥ 2 of 3 cell lineages)*: a) Hemoglobin level <9 g/dL b) Platelets < 100 x 10^3^ /µL c) Neutrophils < 1 x 10^3^/ µL
4. Hypertriglyceridemia and/or hypofibrinogenemia*: a) Fasting triglyceride level of ≥ 3 mmol/L (≥ 265 mg/dL) b) Fibrinogen level of ≤ 150 mg/dL
5. Hemophagocytosis in bone marrow or spleen or lymph nodes, and no evidence of malignancy*
6. Low or absent natural killer-cell activity
7. Ferritin level of ≥ 500 mcg/L*
8. Soluble CD25 level of ≥ 2400 U/mL*

Many triggers for secondary HLH including infectious etiologies have been identified including viral, bacterial, parasitic, and fungal infections. CMV-induced HLH due to immunosuppressive medication has been described in at least one other patient with myasthenia gravis who was being treated with azathioprine. This patient was treated with a combination of dexamethasone, etoposide, and valganciclovir with eventual recovery [9]. From literature review, a case of CMV-induced HLH reported by Divithotawela et al., was treated with anakinra in addition to ganciclovir successfully as a treatment option in a critically ill patient [10]. Treatment of HLH in adults is primarily based on pediatric protocols published by the Histiocyte Society in 1994 (HLH-94) and revised in 2004 (HLH-2004) using dexamethasone and etoposide therapy. Cyclosporin may also sometimes be considered [8,11]. This presents several challenges in the adult population as it may lead to overtreatment and toxicity. Therefore, dose reductions and individualized tailoring of treatment should be considered in each patient. This is particularly important in patients who have underlying rheumatic disease as MAS treatment differs in management, usually involving treatment with pulse dose steroids (1 g/day for three to five consecutive days), as well as IL-1 inhibition with anakinra. IL-6 inhibition with tocilizumab is also being explored [8, 12]. Because our patient had an underlying inflammatory myositis, this led to our decision to recommend anakinra for this adult patient with CMV-induced HLH. Unfortunately, she did not respond to anakinra therapy and etoposide was initiated instead. This highlights that a customized approach may be necessary and individualized treatment depending on patient characteristics is crucial.

## Conclusions

We report a case of a 21-year-old female with inflammatory myositis on azathioprine and prednisone who was diagnosed with CMV-induced HLH treated with anakinra and subsequently etoposide in combination with dexamethasone and antiviral agents. The presentation of HLH can be highly variable and physicians should be familiar with the clinical manifestations as it is often under-recognized. Because HLH can be life-threatening with a high mortality rate, it is important to diagnose and treat HLH early for the most optimal outcomes. Our case highlights that an individualized approach to treatment is important in HLH as underlying patient characteristics may drive response to therapy. 
